# The carboxyl terminus of VEGF-A is a potential target for anti-angiogenic therapy

**DOI:** 10.1007/s10456-014-9444-3

**Published:** 2014-10-02

**Authors:** James G. Carter, Melissa V. R. Gammons, Gopinath Damodaran, Amanda J. Churchill, Steven J. Harper, David O. Bates

**Affiliations:** 1 Microvascular Research Laboratories, School of Physiology and Pharmacology, University of Bristol, Preclinical Veterinary Sciences Building, Southwell Street, Bristol, BS2 8EJ UK; 2 Cancer Biology, Queens Medical Centre, University of Nottingham, D Floor West Block, Nottingham, NG7 2UH UK

**Keywords:** VEGF, Splicing, Bevacizumab, VEGF-A_165_b

## Abstract

Anti-VEGF-A therapy has become a mainstay of treatment for ocular neovascularisation and in cancer; however, their effectiveness is not universal, in some cases only benefiting a minority of patients. Anti-VEGF-A therapies bind and block both pro-angiogenic VEGF-A_xxx_ and the partial agonist VEGF-A_xxx_b isoforms, but their anti-angiogenic benefit only comes about from targeting the pro-angiogenic isoforms. Therefore, antibodies that exclusively target the pro-angiogenic isoforms may be more effective. To determine whether C-terminal-targeted antibodies could inhibit angiogenesis, we generated a polyclonal antibody to the last nine amino acids of VEGF-A_165_ and tested it in vitro and in vivo. The exon8a polyclonal antibody (Exon8apab) did not bind VEGF-A_165_b even at greater than 100-fold excess concentration, and dose dependently inhibited VEGF-A_165_ induced endothelial migration in vitro at concentrations similar to the VEGF-A antibody fragment ranibizumab. Exon8apab can inhibit tumour growth of LS174t cells implanted in vivo and blood vessel growth in the eye in models of age-related macular degeneration, with equal efficacy to non-selective anti-VEGF-A antibodies. It also showed that it was the VEGF-A_xxx_ levels specifically that were upregulated in plasma from patients with proliferative diabetic retinopathy. These results suggest that VEGF-A_165_-specific antibodies can be therapeutically useful.

## Introduction

Angiogenesis is implicated in the pathology of a range of diseases with a vascular element and as a key mediator of blood vessel growth, vascular endothelial growth factor (VEGF) has been extensively studied as a critical protein in pathological neovascularisation [8]. In 2002, the VEGF-A isoform family VEGF-A_xxx_b was discovered and found to possess significant anti-angiogenic properties [3, 25]. These VEGF-A_xxx_b isoforms can be distinguished from the pro-angiogenic VEGF-A isoforms (VEGF-A_xxx_) by their terminal exon selection. Proximal splice site selection in the terminal exon, exon8a, encodes the terminal six amino acid sequence of CDKPRR to create the VEGF-A_xxx_ isoforms, whereas distal splice site selection (exon8b, 66 bp 3′ from exon8a) creates the anti-angiogenic VEGF-A_xxx_b isoforms with an altered 6 terminal amino acid sequence; SLTRKD [13].

Analysis of expression of VEGF-A_xxx_b isoforms shows they form a significant proportion of VEGF-A in most normal, non-angiogenic tissues such as dorsal root ganglia (71 %), lung (82 %) [3], skin (>95 %) [7], prostate [25], colon (>95 %) [24] and vitreous (66 %) [16], but it is a small proportion of the total VEGF-A concentration in angiogenic tissues such as placenta (1.4 %) [4]. Unlike conventional VEGF-A isoforms, VEGF-A_165_b is down-regulated in retinopathy and cancers such as renal cell carcinoma, [3] colon carcinoma [24], prostate carcinoma [25] and malignant melanoma [18].

Furthermore, VEGF-A_165_b expression inhibits VEGF-A_165_ -mediated proliferation, migration and vasodilation in vitro [3, 20, 21] as well as in vivo models of angiogenesis including rat mesentery and rabbit cornea [25], mouse skin and chick chorioallantoic membrane [23], Matrigel implants [10], rat mammary gland [19], rat ovary [1] and tumour models [21, 24].

The ability to detect VEGF-A in biological samples is critical for the assessment of angiogenesis and commercial *pan*VEGF-A ELISA kits are widely available, with the R&D Systems ELISA (DY293B, R&D Systems) one of the most commonly cited. A method for VEGF-A_xxx_b isoform detection in both laboratory and medical samples has already been established [6, 16, 25] and a commercial version has been available for several years (DY3045, R&D Systems). However, similar products to specifically detect VEGF-A_xxx_ isoforms, as opposed to *pan*VEGF, are still relatively scarce. Thus, despite the important physiological role that the balance of VEGF-A_xxx_ and VEGF-A_xxx_b isoforms has in angiogenesis, the ability to distinguish between each isoform family is severely limited experimentally.

The lack of reliable assays is partly due to the success of anti-VEGF-A therapies that bind both VEGF-A_xxx_ and VEGF-A_xxx_b isoforms that, at first glance, do not require a distinction between isoforms. The application of such non-specific therapeutics has been shown to be clinically successful in the treatment of colorectal carcinoma (e.g. bevacizumab, Avastin^®^) [9] and age-related macular degeneration (AMD, e.g. ranibizumab, Lucentis^®^) [11]. However, the application of anti-*pan*VEGF-A therapeutics has no effect on a sub-population of patients [9]. A recent study in metastatic colorectal carcinoma patients showed that only those patients with low VEGF-A_xxx_b:VEGF_total_ isoform ratios respond positively to bevacizumab therapy, whilst those with a higher relative expression of VEGF-A_xxx_b isoforms do not benefit from anti-VEGF-A therapy [2]. Thus, the targeted inhibition of the pro-angiogenic VEGF-A_xxx_ isoforms without the loss of VEGF-A_xxx_b isoforms is a more attractive therapy; inhibiting pathological angiogenesis without the loss of cytoprotective VEGF-A_xxx_b required to maintain a healthy vasculature.

Here, we present data to show that targeted inhibition of VEGF-A_xxx_ isoforms with Exon8apab, a polyclonal antibody specific to the exon8a (CDKPRR) amino acid sequence, is as effective as current anti-*pan*VEGF-A therapeutics but may be more physiologically applicable in the treatment of VEGF-mediated pathologies.

## Methods

### Anti-VEGF-A_165_a antibody

Rabbits were immunized with a nine amino acid peptide encoded by the C-terminal of VEGF-A_165_—TCRCDKPRR—conjugated to KLH by Abgent Inc. Plasma was taken and screened for VEGF-A_165_-specific activity. One rabbit generated specific polyclonal antibodies. Plasma was taken from this rabbit on at least three occasions and activity confirmed. A final bleed was then subjected to immunopurification by standard methodology.

### Cell migration assay

Human umbilical vein endothelial cells (HUVECs) were isolated as previously described and serum starved in endothelial basal media (EBM) for 12 h. Cells were trypsinized and re-suspended in 0.1 % FBS in EBM and 50,000 cells in 500 µl medium were seeded on attachment factor (Cascade Biologics, Portland, OR, USA) coated filter inserts (8 µm pore, 12 mm diameter, Millipore, Billerica, MA, USA) with the treatment (1nM VEGF-A_165_a, with or without VEGF-A antibody, or 10 % FCS as a positive control) in the bottom well. Each treatment was performed in triplicate. Cells were incubated at 37 °C over night. Inserts were washed with PBS and cells fixed with 4 % PFA/PBS pH 7.4 for 10 min. The top of the membrane was carefully cleaned with a sterile cotton bud, the membrane stained with Hoechst 33258 (5 µg/ml in 0.5 % Triton/PBS). Membranes were excised mounted bottom side up on microscope slides with Vectashield (Vetorlabs, Burlingame, CA, USA). Cells were counted in 10 randomly chosen fields away from the edge under a fluorescence microscope (Nikon Eclipse T200). Cell migration was expressed as the number of cells per high power field. The inhibitory effect on migration of VEGF-A antibodies was determined by increasing concentrations of antibody with 1 nM (40 ng/ml) VEGF-A_165_. IC_50_ was calculated from the normalised data using a variable slope sigmoidal fit (Prism4 software).

### Laser-induced choroidal neovascularisation

Six- to eight-week-old C57/B6 mice (B&K Laboratories) were anaesthetised with an intraperitoneal injection of a mixture of 50 mg/kg ketamine and 0.5 mg/kg medetomidine. The pupils were dilated with 2.5 % phenylephrine hydrochloride and 1 % tropicamide. Four photocoagulation lesions were delivered with a krypton red laser (mice: 250 mW, 0.01 s, 75 μm, rats: 200 mW, 0.01 s, 75 μm, IRIS Medical 810 nm Oculight Slx laser) between the “large” retinal vessels in a peripapillary distribution at a distance of 1–2 optic disc diameters in each eye. Only laser lesions with a subretinal bubble at the time of treatment were included in the study. Immediately following laser photocoagulation, the animals received intravitreal injections of 500 ng IgG in the control eye and 500 ng anti-VEGF-A (G6-31) or 500 ng exon8a PAb in the treated eye (day 0 and day 7). Animals were culled on day 14 and eyes fixed, enucleated and choroids stained and examined.

### VEGF-A_xxx_-specific ELISA

Immunoassay 96-well plates were coated with Exon8apab antibody (25 µg/ml in 1× PBS, 100 μl/well) and left overnight at room temperature. After washing in triplicate (0.05 % Tween^®^ in 1× PBS, 200 µl/well), immunoassay plates were blocked (1 % Bovine Serum Albumin in 1× PBS, 200 µl/well) and incubated at 37 °C for minimum of 2 h. The plates were washed and samples added, using recombinant human VEGF-A_165_ (840164, R&D Systems) as a serial dilution control standard. Samples were assessed in triplicate (100 µl/well, diluted in 1 %BSA/PBS). The plate was then incubated at 37 °C for 2 h with shaking.

Following washing, biotinylated goat anti-human *pan*VEGF-A detection antibody (BAF293, R&D Systems) was added at 100 ng/ml in 1 %BSA/PBS (100 µl/well). The plates were then incubated once more at 37 °C for 2 h. Following washing, HRP-conjugated streptavidin was added (1:200 in 1 %BSA/PBS, 100 µl/well, 890803, R&D Systems) and incubated without light exposure or agitation for 30 min at RT. The plates were washed once more, and HRP ELISA substrate (100 µl/well, DY999, R&D Systems) was added before a final incubation without light exposure at RT for 15–30 min. A stop solution (1 M H_2_SO_4_, 50 µl/well) was then added directly to the substrate and resultant colour change measured at 450 nm using an Opsys MR plate reader (Dynex, USA).

### Plasma extraction and analysis

Patients were recruited from the vitrectomy clinics from the Bristol Eye Hospital. All study participants were Caucasians of Northern European origin. Ethics approval for the study was obtained from the North Somerset and South Bristol Research Ethics Committee and protocols conformed to the tenets of the Declaration of Helsinki, as revised in 2000. A venous blood sample was obtained from each participant after informed written consent. Patient samples were divided by diabetic status into three subgroups; proliferative diabetic retinopathy (PDR group, *n* = 8), patients with non-proliferative diabetic retinopathy (NPDR group, *n* = 11) and patients without diabetes (non-diabetic/ND group, *n* = 22). Peripheral blood plasma was separated by centrifugation (15 min at 2,000×*g*) and aliquoted into individual vials (100–200 µl) for storage at −80 °C prior to the study. Assessment of VEGF-A_xxx_a isoform concentrations were determined using the ELISA protocol above, whereas VEGF-A_xxx_b isoforms were assessed by ELISA as described in [6].

### Tumour growth assays

2 × 10^6^ LS174t colon cancer cells were prepared after trypsinisation in 0.2 ml PBS and injected subcutaneously in the dorsum of 6 nude Balb/C mice for each group. The mice were monitored bi-weekly for tumour development. Once tumours had developed mice were randomly allocated to each of three groups and injected i.p. with either 50 µg bevacizumab, Exon8apab or mouse IgG in 100 µl saline, coded and blinded for the injector and measurer. Tumours were measured 3 days later and injected with a second dose of the antibody. Animals were killed 7 days later, because insufficient Exon8apab antibody was available for further treatment.

## Results

### Exon8apab is selective for VEGF-A_165_ over VEGF-A_165_ b

**Fig. 1:**
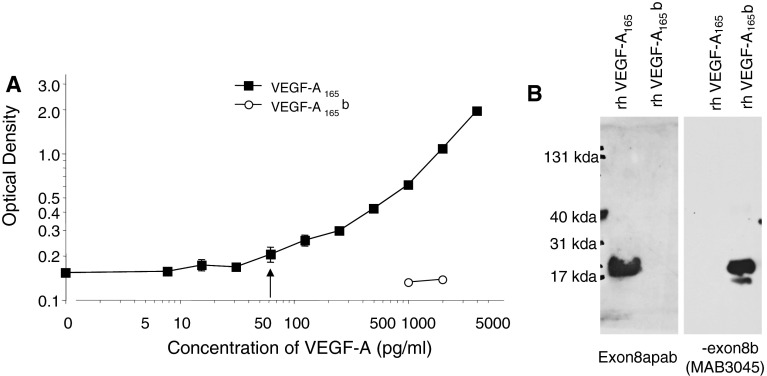
Exon8apab is specific for VEGF-A_165_a. **a** An ELISA with Exon8apab as a capture antibody and a biotinylated goat anti-human panVEGF-A detection antibody with increasing concentration of VEGF_165_a (*closed circles*) or VEGF_165_b (*open circles*) was carried out. **b** Western blot using the two antibodies demonstrated specificity of the Exon8apab for 20 ng recombinant human VEGF-A_165_a and a monoclonal antibody to the c terminus of rhVEGF-A_165_b for 20 ng of VEGF-A_165_b. Neither antibody recognised the other protein

Specificity of the antibody for the angiogenic phenotypes was determined by ELISA (Fig. 1a) and Western blot (Fig. 1b). Recombinant human VEGF-A_165_ but not VEGF-A_165_b was detected by Exon8apab. The commercially available VEGF-A_xxx_b-specific antibody MAB3045 (R&D Systems) in the same assays detected VEGF-A_165_b but not VEGF-A_165_. Using Exon8apab as a capture antibody for VEGF-A protein in a sandwich ELISA assay showed detection above background at 62.5 pg/ml (similar to commercially available pan-VEGF-A antibodies), In contrast recombinant human VEGF-A_xxx_b was not detected using Exon8apab even up to 2000 pg/ml.

### Exon8apab inhibits endothelial cell migration in response to VEGF-A_165_

**Fig. 2:**
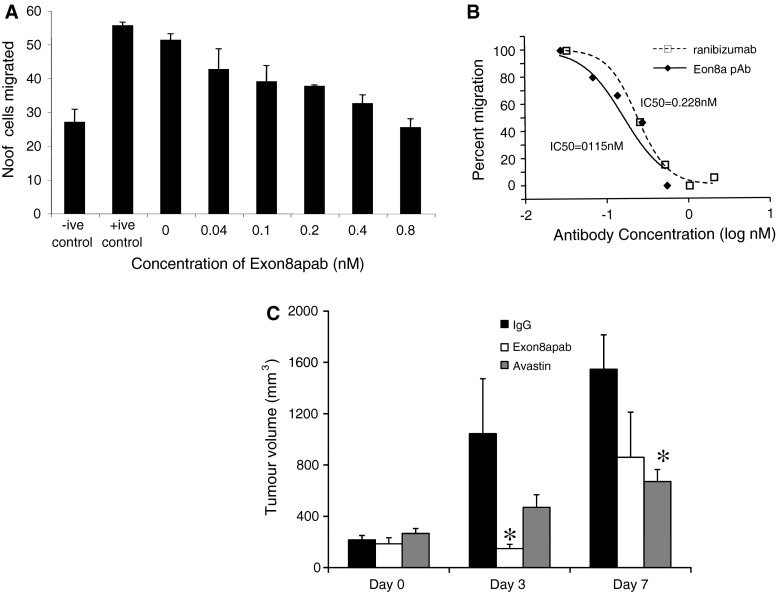
Exon8apab inhibits angiogenesis. **a** Endothelial cells were seeded onto polycarbonate filters of transwell inserts. Increasing concentrations of Exon8apab and 40 ng/ml rhVEGF-A_165_a was added to the lower well. Cell migration across was measured by counting cells on the lower side of the membrane 24 h after seeding. **b** The experiment was repeated using either Exon8apab or ranibizumab. IC_50_ was calculated using a variable slope dose inhibition curve (Prism4.0). **c** LS174t cells (1 × 10^9^) were implanted into nude mice and tumours allowed to grow to approximately 200 mm^3^. Animals were then treated with 2 mg/kg Exon8apab antibody at D0 and treatment repeated at D3. Tumour volumes were measured once more at D7. * *p* < 0.05, one-way ANOVA, Bonferroni test

Treatment of HUVEC cells with VEGF-A_165_ resulted in a significant migration similar to that induced by 10 % serum (Fig. 2a). This was dose dependently inhibited by treatment with Exon8apab (IC_50_ = 0.115nM 95 % CI 0.07–0.19, Fig. 2b). Comparison with ranibizumab demonstrated that the potency of the polyclonal antibody was greater, but not statistically significantly so than that of a *pan*VEGF-A antibody (IC_50_ = 0.228, 95 % CI 0.17–0.311, *p* = 0.059).

### Exon8apab slows tumour growth similarly to anti-VEGF-A antibodies

To determine whether the antibody could inhibit tumour growth, we treated LS174t heterotopic colon cancer tumour bearing mice with bevacizumab or anti-VEGF-A_165_ Exon8apab (*n* = 6 per group). Tumours were not different in size after 1 week of implantation due to matched allocation to treatment group. Three days after initial treatment Exon8apab treated tumours were significantly smaller than IgG treated tumours (Fig. 2c). After 7 days, tumours from bevacizumab-treated mice groups were smaller than the IgG controls, (*p* < 0.05, one-way ANOVA). Whilst 5/6 mice treated with Exon8apab showed tumours comparable with the bevacizumab treatment group, the remaining tumour grew significantly in those 4 days. As a result, whilst mean tumour volume was comparable with bevacizumab, there were no statistically significant differences between exon8apab treatment and IgG controls at Day 7.

### Exon8apab inhibited choroidal neovascularisation

**Fig. 3:**
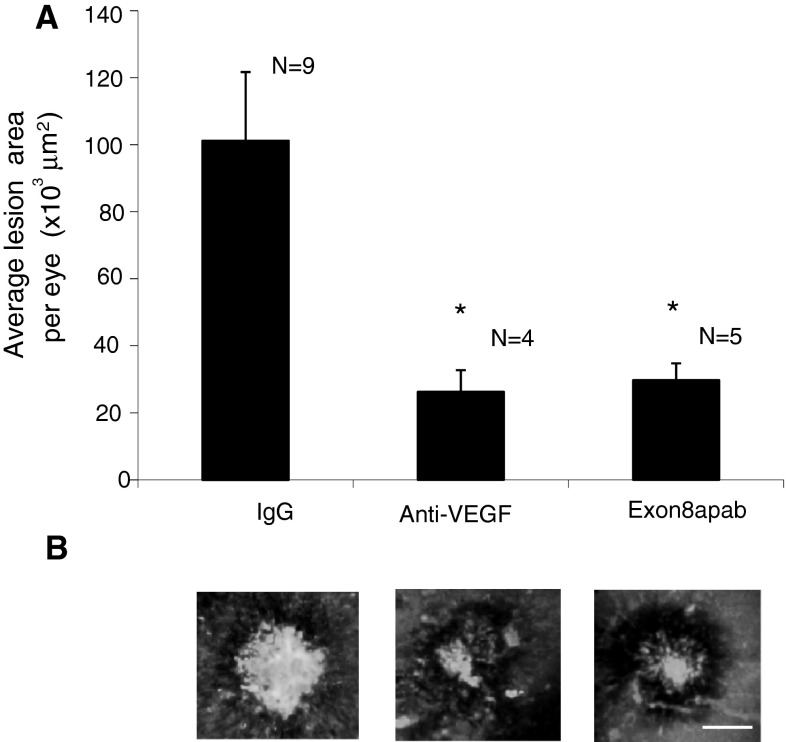
Exon8apab inhibits laser-induced CNV in mice. C57/B6 mice underwent retinal lasercoagulation in both eyes (IRIDEX Oculight GLX λ—810 nm, 250 mV, 0.1 s, 75 μm, 4 lesions/eye). Animals were subjected to intraocular injection with either 500 ng (250 ng/µl) of an anti-mouse VEGF neutralising antibody, and control eyes injected with either mouse IgG, or 500 ng (250 ng/µl) of Exon8apab with a control rabbit IgG—controls administered at the same concentration, directly after laser procedure (D0) and on day 7. Staining with isolectin-B4 of the RPE-choroid-sclera complex showed a decrease in lesion size for anti-VEGF and Exon8apab (*N,* number of eyes; * *p* < 0.05, one-way ANOVA, Bonferroni post hoc). Merged lesions were excluded from the study. *Scale bar* 50 µm

As the tumour studies required substantial amounts of the antibody, we used a laser-induced choroidal neovascularisation (CNV) assay to determine the anti-angiogenic activity of Exon8apab. In this model of angiogenesis, intraocular treatment with Exon8apab (250 ng/µl) had similar inhibitory effects to an anti-mouse VEGF-A antibody (250 ng/µl), with reduced lesion size at 7 days post-photocoagulation (*p* < 0.05, one-way ANOVA, Bonferroni post hoc) (Fig. 3).

### Exon8apab detects circulating VEGF-A_165_a

We used Exon8apab to detect circulating VEGF-A levels in plasma from patients with diabetes. Non-diabetic patients (ND), patients with non-proliferative diabetic retinopathy (NPDR) and proliferative diabetic retinopathy patients (PDR) had their venous blood plasma assessed for VEGF-A_xxx_b using an ELISA method adapted from [6] and VEGF-A_xxx_ using Exon8apab. Of the 45 patients from which plasma was collected VEGF levels were undetectable (<15 pg/ml) in 7. The remaining 83 % of patients had plasma levels detected by either VEGF-A_165_b alone (2 patients), or both isoforms (32 patients).

**Fig. 4:**
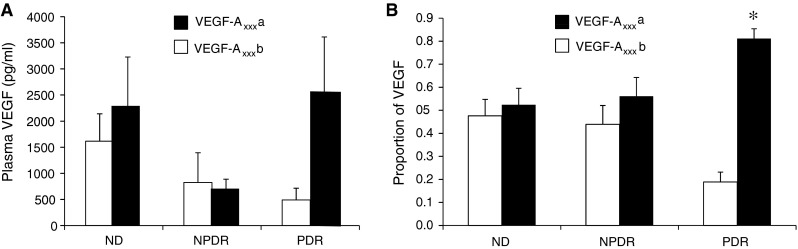
Exon8apab measures VEGF levels in human plasma. VEGF levels were measured in plasma from 32 patients, 5 of whom had proliferative diabetic retinopathy (PDR), 11 non-proliferative diabetic retinopathy (NPDR) and 18 control patients with no diabetes (ND). **a** VEGF concentrations were measured by ELISA using either the anti-VEGF-A_165_b as a capture antibody, or exon8apab as a capture antibody. **b** The relative amount of the two isoforms was calculated as a per cent of the total—e.g. 100*VEGF-A_xxx_b/(VEGF-A_xxx_a + VEGF-A_xxx_b). * *p* < 0.05 compared with VEGF-A_xxx_b, Bonferroni

Assessment of VEGF-A concentrations in plasma showed no statistically significant differences between the different subgroups by VEGF-A_xxx_ and VEGF-A_xxx_b assessment (*p* > 0.05, *t* test, Fig. 4a). However, there did appear to be a trend with higher anti-angiogenic VEGF-A_xxx_b in the ND/NPDR groups and higher pro-angiogenic VEGF-A_xxx_ in the PDR subgroup. When the proportion of VEGF-A isoforms to combined total (VEGF-A_xxx_ + VEGF-A_xxx_b = VEGF_sum_) was calculated, there appears to be a consistent shift from VEGF-A_xxx_b predominating in non-diabetics in favour of pro-angiogenic VEGF-A_xxx_ in the PDR group (Fig. 4b, *p* = 0.050 chi-squared test for trend). Analysis within subgroups showed that non-diabetic patients have no difference in the proportion of VEGF-A_xxx_b (47.6 vs. 52.4 %). The non-proliferative diabetics appear to show an intermediary balance of VEGF-A_xxx_b to VEGF-A_xxx_ (43.9 vs. 56.1 %), whereas in proliferative diabetic patients the majority of VEGF-A produced is VEGF-A_xxx_ (81.1 vs. 18.9 % VEGF-A_xxx_b, *p* = <0.01 one-way ANOVA, Bonferroni test.).

## Discussion

Here, we show that an antibody directed against the C terminus of VEGF-A_165_ is able to inhibit VEGF-A_165_-mediated cell migration, angiogenesis and tumour growth in vivo and can be used to detect VEGF-A_165_ (but also presumably other VEGF-A_xxx_ isoforms) in human plasma. The antibody generated was a polyclonal antibody from a single rabbit. We attempted during this project to generate monoclonal antibodies from mice both in house and commercially and failed on three occasions. Moreover, only one of six rabbits generated antibodies that were effective in detecting VEGF-A_165_. The supply of the antibody is therefore limited, and we surmise that antigenicity of the peptide is relatively low. Interestingly, there have only ever been two published antibodies against the C terminus of VEGF-A_165_b—this one and the original VPF antibody generated by Donald Senger in 1986 [22]. Both are rabbit polyclonals, and all other VEGF antibodies commercially available, or available by collaboration have targeted either exons 3–4 or exon 6 [17]. It is therefore clear that generation of C-terminal antibodies is not widely in use, and we have not been able to generate specific antibodies with the efforts described here. For this reason, only a preliminary tumour study was carried out in mice, but the evidence we have suggests that C-terminal antibodies targeting all VEGF-A_xxx_a isoforms, when they can be generated, are at least as effective as receptor binding domain antibodies such as ranibizumab or bevacizumab at inhibiting VEGF-A_165_.

These results raise a number of interesting questions. First, it shows that it is possible to generate antibodies that specifically target the pro-angiogenic isoforms of VEGF, without affecting the anti-angiogenic, cytoprotective isoforms such as VEGF-A_165_b. This would result in antibodies that do not suffer from the resistance associated with targeting all VEGF isoforms in colon cancer for instance [2]. It may also result in safer, more effective anti-VEGF-A therapies for eye disease, where long-term use of *pan*VEGF-A antibodies are associated with progressive vision loss, possibly due to geographic atrophy [14].

These antibodies also raise the interesting likelihood that conventional assessment techniques for measuring circulating VEGF-A levels are inaccurate and could be replaced. There are several limitations to the application of these *pan*VEGF-A assays. First, they are not applicable to all sample types. For example, competition for epitopes between antibodies and native molecules such as high concentrations of the proteinase inhibitor α_2_-macroglobin in human plasma are capable of binding VEGF-A and therefore reduces anti-VEGF-A antibody binding by 82 % [5]. Secondly, the inability for *pan*VEGF-A detection methods to distinguish between VEGF-A_xxx_ and VEGF-A_xxx_b isoforms means any alterations in splicing with significant physiological implications will not be detected. Thirdly, such *pan*VEGF-A antibodies have different affinities for the different isoform families. Assessment by both ELISA and surface plasmon resonance showed that the R&D Systems DuoSet *pan*VEGF-A antibody only detects 42 ± 0.4 % of VEGF-A_xxx_b, due to a difference in binding affinity; 602 pM for VEGF-A_xxx_ versus 3.98 nM for VEGF-A_xxx_b, an ~6.6-fold difference in affinity [24]. The results of such assays mean that the circulating levels of VEGF-A found in normal plasma are regularly reported at variously between 0 and 200 pg/ml, whereas early assays using enzyme immunoassays (EIA) or differential epitope antibodies put the concentration at >1 ng/ml. Here, we show that in normal control patients the total VEGF-A levels are around 4 ng/ml, of which 1.5 ng/ml is VEGF-A_xxx_ and 2.5 ng/ml is VEGF-A_165_b. This fits with early methods, and with Western blots showing detectable levels of VEGF-A in plasma when concentrated 10× (unpublished data), suggesting that the original concentration of the order of 1–10 ng/ml (200 pg/ml, even concentrated 10x would require 1 ml of plasma to be loaded into a single well of a SDS PAGE gel).

The results also suggest that the C-terminal tail of VEGF-A_165_ is required for VEGFR activation. However, crystallographic studies of VEGF-A have shown that it is the amino acids encoded by exons 3 and 4, not exon 8, that are the receptor binding domain [12]. Whilst the full length VEGF including the C-terminal tail has never been crystallised, and it has been shown that VEGF-A_159_ (a protein missing the last six amino acids) is capable of both receptor binding and inducing angiogenesis [23]. Crucially, despite equal receptor binding affinity between VEGF-A_165_, VEGF-A_159_ and VEGF-A_165_b, the C-terminal modulation determines whether the response is angiogenic (receptor activation) or anti-angiogenic (inhibitory). If the last few (>6) amino acids of the protein were to be required to interact with the receptor binding domain then an antibody to the C terminus could disrupt that activation. In addition, recent studies of the VEGFR co-receptor neuropilin-1 (Nrp-1) have shown that the C terminus of VEGF-A_165_, particularly Arg-164, mediates high affinity binding of Nrp-1, increasing VEGFR activity [15]. These results therefore suggest that the C terminus of the VEGF-A protein might be able to interact with the receptor binding domain, or at least be brought within its vicinity during receptor activation. Thus, the action of the Exon8apab antibody must be interfering with the C terminus-neuropilin/receptor binding domain interaction, presumably through disruption to stoichiometric interaction.

It is clear that the work described here is preliminary, in that significant amounts of antibody are not yet available and monoclonal antibodies have not been produced, so the effects on VEGFR phosphorylation, tumour angiogenesis, or even longer term analysis of the effects on tumour growth are yet to be deduced. However, these preliminary data indicate that VEGF-A_xxx_-specific antibodies may have considerable benefit both for research and diagnostically in certain patients. Furthermore, VEGF-A_xxx_-specific antibodies may provide the basis for a highly targeted and thus more widely effective therapeutics than current anti-VEGF therapies.
